# What we can see from very small size sample of metagenomic sequences

**DOI:** 10.1186/s12859-018-2431-8

**Published:** 2018-11-03

**Authors:** Jaesik Kwak, Joonhong Park

**Affiliations:** 1 Graduate Program in Technology Policy, Yonsei University, 50 Yonsei Ro, Seodaemun Gu, Seoul, 038722 South Korea; 2 School of Civil and Environmental Engineering, Yonsei University, 50 Yonsei Ro, Seodaemun Gu, Seoul, 038722 South Korea

**Keywords:** Metagenomics, Sampling, Mock community, MG-RAST, BLAST

## Abstract

**Background:**

Since the analysis of a large number of metagenomic sequences costs heavy computing resources and takes long time, we examined a selected small part of metagenomic sequences as “sample”s of the entire full sequences, both for a mock community and for 10 different existing metagenomics case studies. A mock community with 10 bacterial strains was prepared, and their mixed genome were sequenced by Hiseq. The hits of BLAST search for reference genome of each strain were counted. Each of 176 different small parts selected from these sequences were also searched by BLAST and their hits were also counted, in order to compare them to the original search results from the full sequences. We also prepared small parts of sequences which were selected from 10 publicly downloadable research data of MG-RAST service, and analyzed these samples with MG-RAST.

**Results:**

Both the BLAST search tests of the mock community and the results from the publicly downloadable researches of MG-RAST show that sampling an extremely small part from sequence data is useful to estimate brief taxonomic information of the original metagenomic sequences. For 9 cases out of 10, the most annotated classes from the MG-RAST analyses of the selected partial sample sequences are the same as the ones from the originals.

**Conclusions:**

When a researcher wants to estimate brief information of a metagenome’s taxonomic distribution with less computing resources and within shorter time, the researcher can analyze a selected small part of metagenomic sequences. With this approach, we can also build a strategy to monitor metagenome samples of wider geographic area, more frequently.

**Electronic supplementary material:**

The online version of this article (10.1186/s12859-018-2431-8) contains supplementary material, which is available to authorized users.

## Background

As next-generation sequencing is getting popular [1], a large number of genome sequences now can be easily generated for metagenomics research [2]. However, since analyzing a large number of sequences usually costs heavy computing resources and takes long time [3].

To shorten computation time and reduce requirements for computing resources, researchers introduced advanced algorithmic techniques and database optimization methods. MetaPhlAn uses a database engineered to contain specific marker genes to do sequence classification quickly [4]. Kraken searches a large k-mer database designed for its own search method to look up its taxonomic trees [5]. Centrifuge focuses more on compression of database sequences to reduce the size of database to search [6].

On the other hand, there have been several different ways to get information only from a relatively small part of the available data [7].

One example to reduce the cost of sequencing and computing was a study to get an optimal depth of sequencing for16s rRNA [8]. This study demonstrated that a small number of Illumina single-end reads, such as 2000 reads, were enough to recapture the taxonomy information and diversity patterns. It showed a possibility that meaningful information can be derived even from a small portion of full sequences. However, it was tested only for a certain type of gene, 16S rRNA [9].

Another example was “genome skimming” study that showed the simulation results of rDNA assembly from shallow sequencing of plant genomes [10]. Based on the efficiency of the shallow sequencing that identified the low-copy fraction of the nuclear genome, this study suggested a strategy, where there are multiple candidate species of interest, using shallow sequencing to choose a species with the best condition, before using deeper sequencing of that chosen species to know more details.

This concept of genome skimming is also applicable to metagenome. One study pointed out that “metagenome skimming” can be an efficient tool to capture “the genomic diversity of poorly studied, species-rich lineages”, after analyzing the sequencing results on two pools of Coleopteran, that consisted about 200 species [11]. However, both studies targeted eucaryocyte and used assembly method to analyze taxonomy, that still requires long computation time for assembling process and a large amount of sequences, which were more than hundred thousands of reads.

### Aims and objectives

In this study, getting taxonomic information from small size sample of a large metagenome sequence data was examined, in order to save computing resources and to shorten processing time.

We utilized a simple rarefaction technique, often used for various studies such as determination of optimal sequencing depth [12]. We applied it to estimating brief taxonomic information from extremely small parts of various metagenomic sequences. We wanted to find out how realistic that the extraction of taxonomic information from those small parts is in practical cases. If it is a practical approach, we might develop a protocol or a standard to preview or pre-check metagenomic sequences with a quick estimation before doing a full-scale analysis for them.

We selected a small part of metagenomic sequences in several ways. We treated these selected sequences as a sample of original full sequences. The phylum and the class with dominant populations were annotated in the sample and compared to ones annotated in the original full sequences, since they are generally considered as important information in metagenomics [13]. The diversities of phyla and classes were also compared.

A mock community, which was intentionally made of known bacterial strains to get a mixed genome, was examined with BLAST search [14]. Since we know the real taxonomic information of the mock community, we can evaluate how well the samples that we made represent the original taxonomic information. We also applied this approach to known results of existing researches, which are available publicly in MG-RAST web site, which has been an open access web service widely used for metagenomics analysis [15].

## Results

### Mock community

The original full sequences obtained from the mock community of 10 strains were about 1,220,000 reads or 12.3 Gb. The GC content calculated from them was 53.1%.

**Fig. 1:**
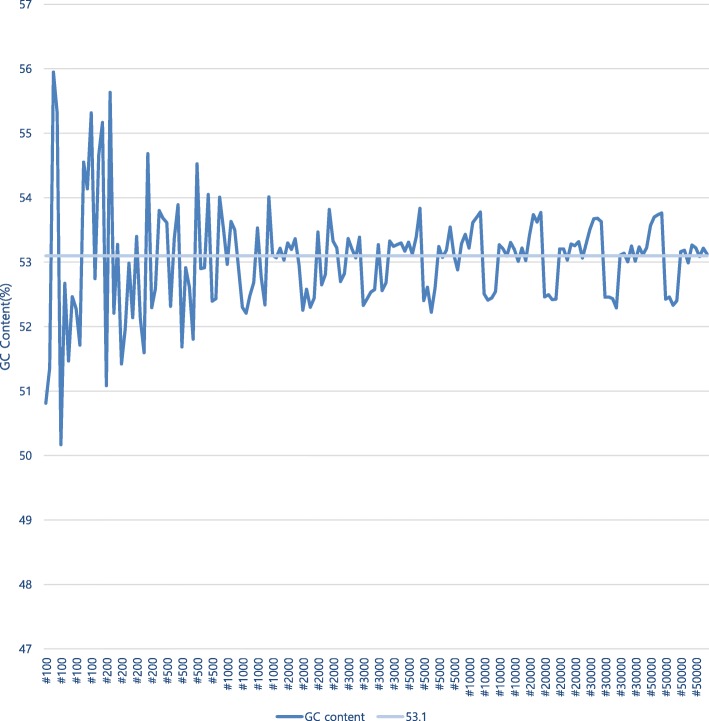
GC content of samples (The labels of x-axis mean the sample sizes. They are placed per one selection type. This means a label represents 4 samples made by 4 different K numbers)

The results of the GC content calculation for 176 different samples, which were selected from the original full sequences by 16 different selection types for each of 11 different sample sizes from 100 to 50,000 reads, show that GC content values get closer to 53.1%, as the sizes of the samples increase. (Fig. 1) This can be regarded as supportive evidence that a sample with a large enough size represents the nature of the original full sequences.

To analyze the taxonomy of this mock community, the numbers of hit reads from BLAST search for each of 10 strains of the original full sequences were counted. Their ratios to the sum of all 10 strains’ hits range from 0.014 to 0.186. (Table 1) These are the original values that we want to estimate with BLAST searches.

**Table 1:** Hits of BLAST searches in the original full sequences of the mock community

Strain	Number of Hits	Ratio: Number of hits for each strain/Sum(=164,662,612)
*Escherichia coli* KCTC 2571	30,658,032	0.1862
Escherichia coli Strain W	29,390,176	0.1785
*Staphylococcus epidermidis* ATCC	18,862,322	0.1146
*Pseudomonas stutzeri* ATCC 17588	18,559,245	0.1127
*Klebsiella pneumoniae* KCTC 2242	15,708,328	0.0954
Chromobacterium violaceum ATCC 12472	15,466,319	0.0939
Polaromonas naphthalenivorans CJ2	14,081,217	0.0855
Corynebacterium glutamicum ATCC 13032	10,351,377	0.0629
Roseobacter denitrificans OCh114	9,332,359	0.0567
Arthrobacter chlorophenolicus A6	2,253,237	0.0137
Sum	164,662,612	1

In order to do the estimation, the ratio of hit reads counted for each strain to the sum of all 10 strains’ hits was calculated for each of the 176 different samples, again, which were selected from the original full sequences by 16 different selection types per 11 different sample sizes.

**Fig. 2:**
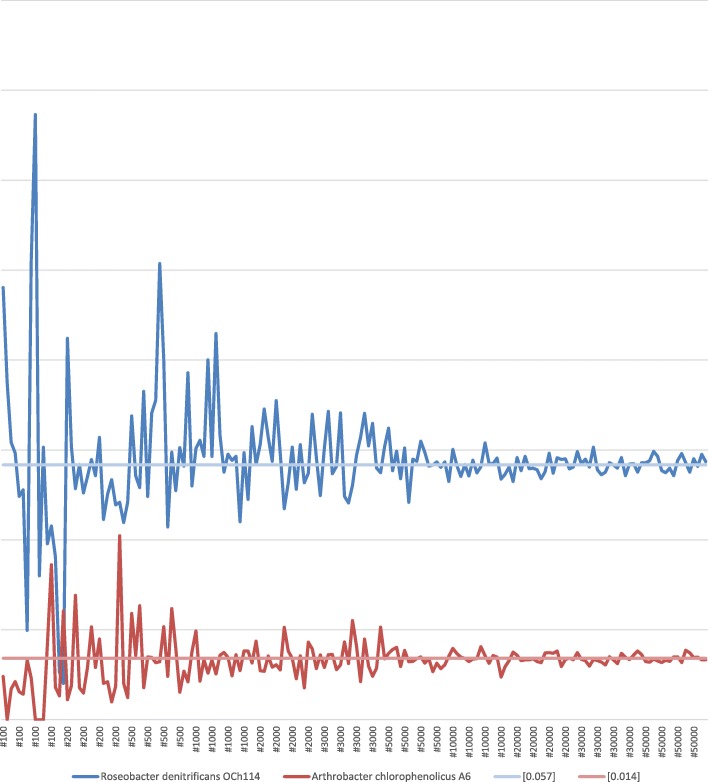
<Hit for strain/sums of all Hits> for each sample (The labels of x-axis mean the sample sizes. They are placed per one selection type. This means a label represents 4 samples made by 4 different K numbers)

The result of the calculation from the samples shows that the ratio values for Roseobacter get closer to 0.057, which was the ratio value of Roseobacter calculated from the original full sequences, as the sizes of the samples increase. The ratio values for Arthrobacter get closer to 0.014 similarly. (Fig. 2) The ratio values calculated for the samples of the other strains also show the similar results. To show the tendency that the deviation from the different sampling methods decreases while the size of the sample increases, the smallest values (Additional file 1: Table S1) and the largest values (Additional file 1: Table S2) among the ratio values calculated from 16 samples of each sample size were tabulated. The standard deviation values out of the ratios calculated from 16 samples of each sample size were also tabulated. (Additional file 1: Table S3).

As the size of the sample increases, the smallest values and the largest values show their tendency of getting closer to the ratio values calculated from the original full sequences. At the same time, as the size of the sample increases, the standard deviation value mainly decrease, though there are a few exceptions, since there are relatively large statistical errors where the values are small.

Again, these results support the tendency that a sample with a large enough size has its hit ratios that are close to ones of the original full sequence.

This means that small part of the original full sequences can be used to estimate original taxonomic annotation regardless of selection type, especially for relative comparison, such as to answer a question of which class is annotated most, and a question of which phylum is more annotated than another phylum.

Meanwhile, we can explain the difference between the results from the original full sequences and the ones from the samples as a general statistical error problem of a small size sample.

For a given margin of error, we can approximate a proper sample size, if we consider that estimating a taxonomic proportion of sequences is similar to a general statistical sampling problem, such as a poll to estimate a proportion of voters to an election candidate.

For example, as a rough approximation, if we assume that a given unknown set of metagenomic sequences follows a normal distribution and expected proportion of reads classified as a certain taxon is close to 1/2, which is a widely used value where we do not have any initial information about the actual proportion and the start-up cost of sampling is expensive [16], there is a simplified equation to calculate the size of the sample for a margin of error. (Eq. 1.) [16] By this calculation, the sample size for 1% margin of error and 85% confidence is about 5000 (5184).

$$ \mathrm{n}=\frac{{\left(\mathrm{Z}\upalpha /2\right)}^2\kern0.5em \cdot \frac{1}{2}\cdot \frac{1}{2}}{{\mathrm{E}}^2} $$

Eq. 1. Determining the sample size n in estimation of population proportion, where the probability of the range greater than Zα/2 at the standard normal distribution equals to (1-*confidence*)/2, and E is margin of error.

**Fig. 3:**
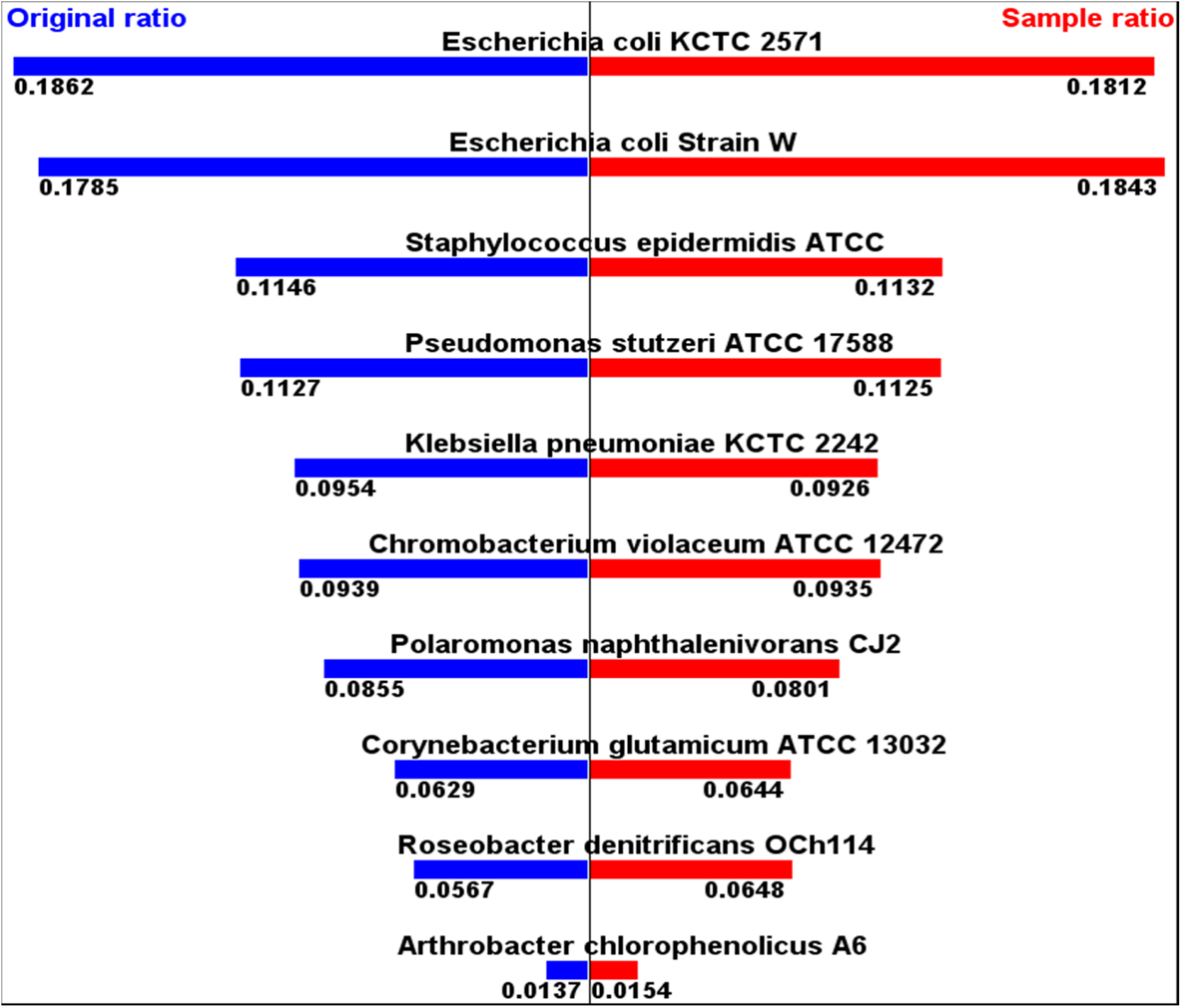
<Hits of strain/sums of all hits> from original and from sample with 5000 Reads

If we apply this margin of error calculation to the mock community test, the result from this margin of error calculation might be smaller than the actual errors, because all the ratio values of the mock community from the original full sequences are smaller than 1/2. Nevertheless, BLAST search result from a sample made by selecting 5000 reads from the start of the original full sequences (“selection type 1” and 0 as “K number”) of this mock community still gives fair estimation of the ratio values. (Fig. 3).

We can compare this to a more general case of statistical sampling problem. For instance, we made the sample whose size is 5000 reads to estimate total 1.22 million reads. On the other hand, New York Times/CBS News performed a poll of 1426 people for 2016 U.S. Presidential election of total 137 million voters [17].

### MG-RAST: Applicability in full-scale metagenomics sequencedata sets

All the GC content values calculated from the original full sequences of the 10 public MG-RAST projects, that all have more than 170,000,000 reads, were compared to the GC content values calculated from the samples, that only have 5000 reads selected from them. (Table 2) In most cases, the GC content values calculated from the samples estimate the ones calculated from the originals well.

**Table 2:** GC Contents, original vs. sample

Original MG-RAST ID	Sample MG-RAST ID	Original GC Content (%)	Sample GC Content (%)
4,539,528.3	4,701,886.3	62.975	62.161
4,510,219.3	4,701,884.3	53.813	51.365
4,510,173.3	4,701,887.3	50.512	50.477
4,509,400.3	4,701,883.3	62.269	62.468
4,562,385.3	4,701,888.3	56.816	57.733
4,538,997.3	4,701,892.3	58.078	56.902
4,539,575.3	4,701,885.3	60.177	60.568
4,587,432.3	4,701,891.3	52.943	52.68
4,555,915.3	4,701,890.3	48.254	48.132
4,533,611.3	4,701,889.3	56.184	45.012

The most annotated phyla and classes from the original MG-RAST research data were compared to the ones of the samples. (Table 3) For 9 cases out of 10, the most annotated phyla from the MG-RAST projects of the samples show the same results as the ones of the original data. For 9 cases out of 10, the most annotated classes are the same between the original MG-RAST research data and the ones of the samples. Considering that 4 different classes were shown among all the cases, these 9 out of 10 matches support the assumption that these samples can estimate the brief taxonomic information of the originals.

**Table 3:** Most annotated phylum and classes, original vs. sample

Original MG-RAST ID	Sample MG-RAST ID	Most Annotated Phylum of Original	Most Annotated Phylum of Sample	Most Annotated Class of Original	Most Annotated Class of Sample
4,539,528.3	4,701,886.3	Proteobacteria	Proteobacteria	Actinobacteria (class)	Actinobacteria (class)
4,510,219.3	4,701,884.3	Proteobacteria	Proteobacteria	Deltaproteobacteria	Deltaproteobacteria
4,510,173.3	4,701,887.3	Proteobacteria	Proteobacteria	Gammaproteobacteria	Gammaproteobacteria
4,509,400.3	4,701,883.3	Proteobacteria	Proteobacteria	Actinobacteria (class)	Actinobacteria (class)
4,562,385.3	4,701,888.3	Proteobacteria	Proteobacteria	Gammaproteobacteria	Gammaproteobacteria
4,538,997.3	4,701,892.3	Proteobacteria	Proteobacteria	Alphaproteobacteria	Deltaproteobacteria
4,539,575.3	4,701,885.3	Proteobacteria	Proteobacteria	Alphaproteobacteria	Alphaproteobacteria
4,587,432.3	4,701,891.3	Firmicutes	Actinobacteria	Actinobacteria (class)	Actinobacteria (class)
4,555,915.3	4,701,890.3	Ascomycota	Ascomycota	Gammaproteobacteria	Gammaproteobacteria
4,533,611.3	4,701,889.3	Proteobacteria	Proteobacteria	Alphaproteobacteria	Alphaproteobacteria

**Fig. 4:**
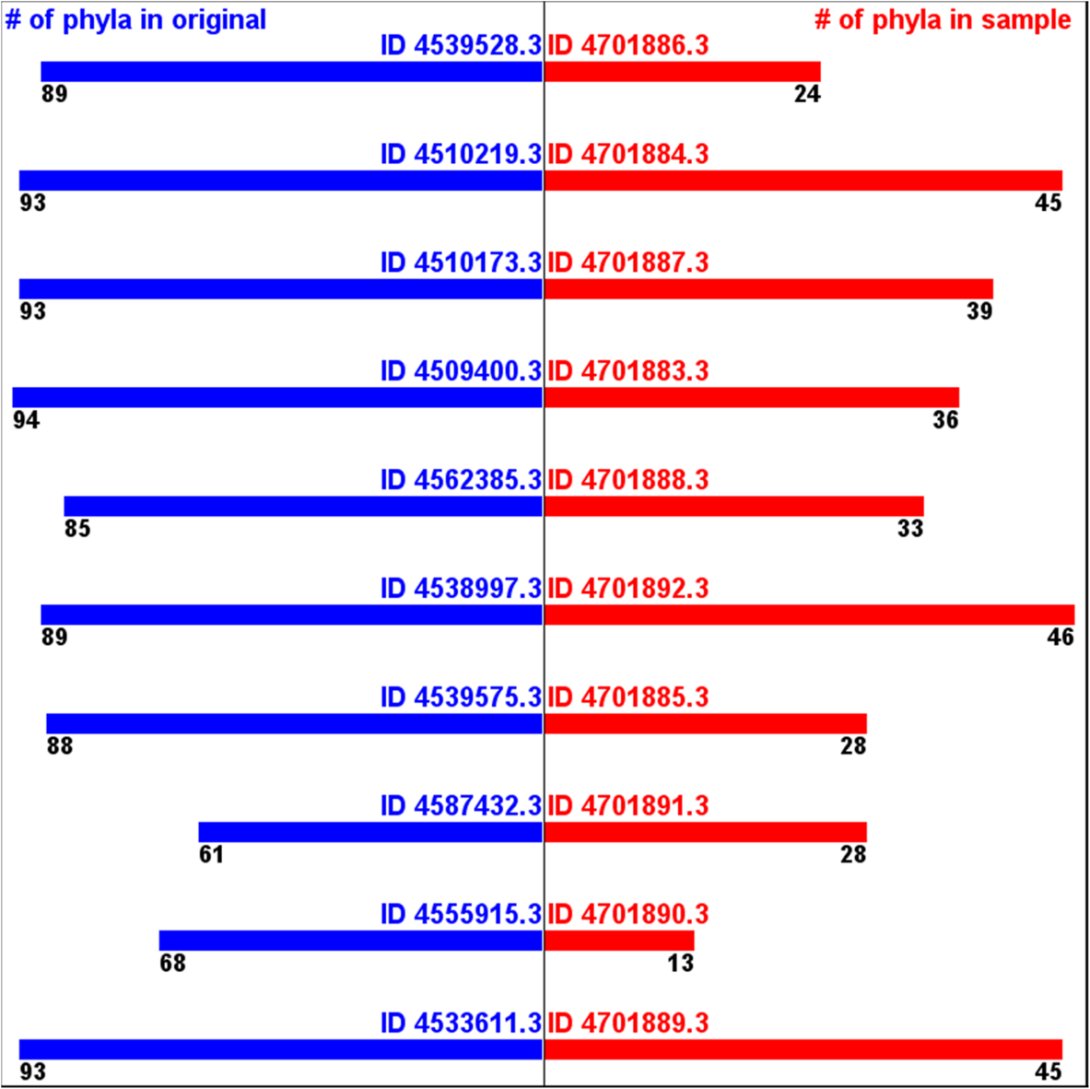
Numbers of annotated phyla -originals vs. samples

**Fig. 5:**
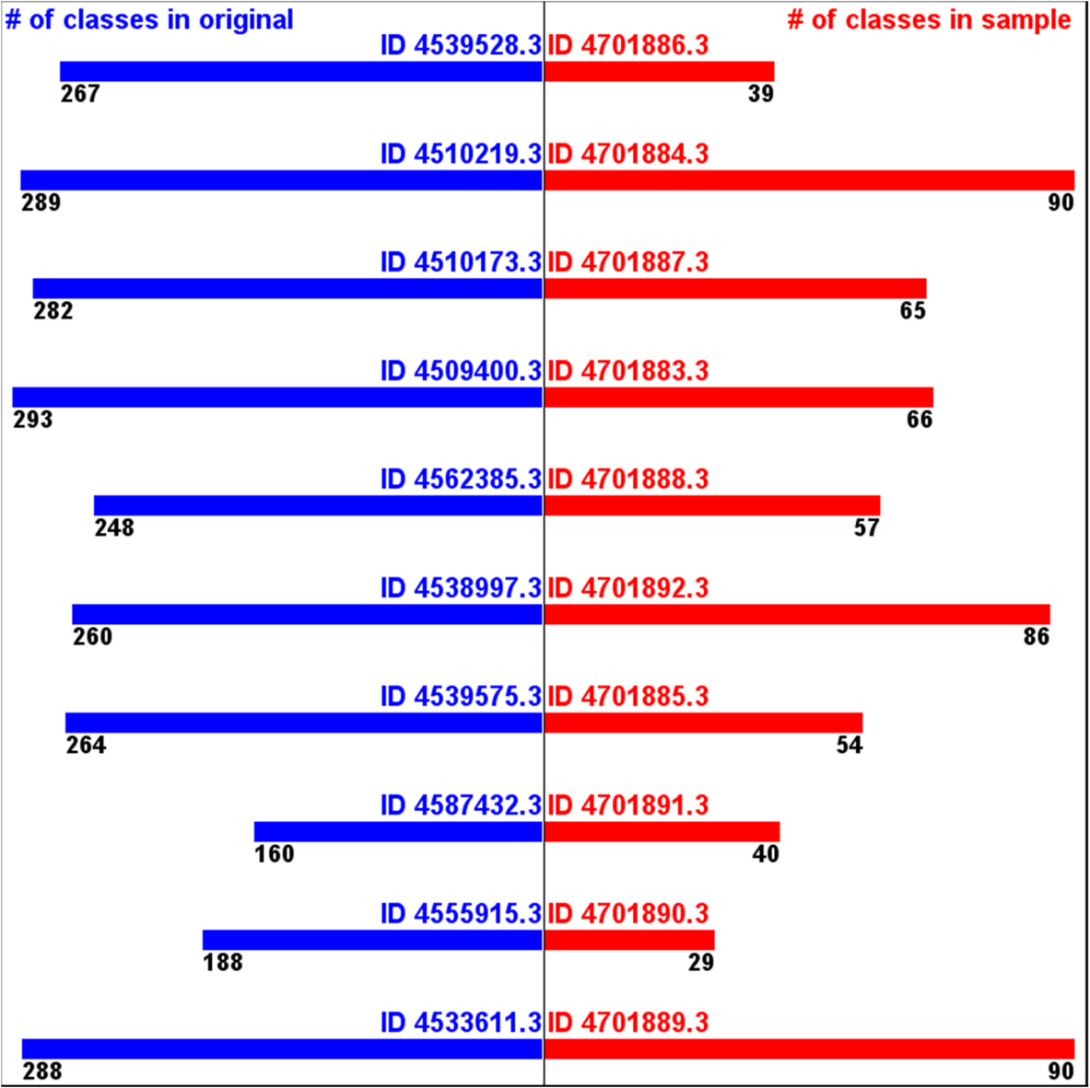
Numbers of annotated classes -originals vs. samples

On the other hand, the numbers of the annotated phyla from the samples tend to be smaller than the ones from the originals. (Fig. 4) The numbers of the annotated classes from the samples tend to be even much smaller than the ones from the samples. (Fig. 5) These are because the samples did not include different sequences representing all the different phyla and classes in the original data. A phylum or a class that presents only a small number of sequences in original has low probability of being captured in a sample. This implies that this type of sampling cannot take all the taxonomic diversity information.

**Fig. 6:**
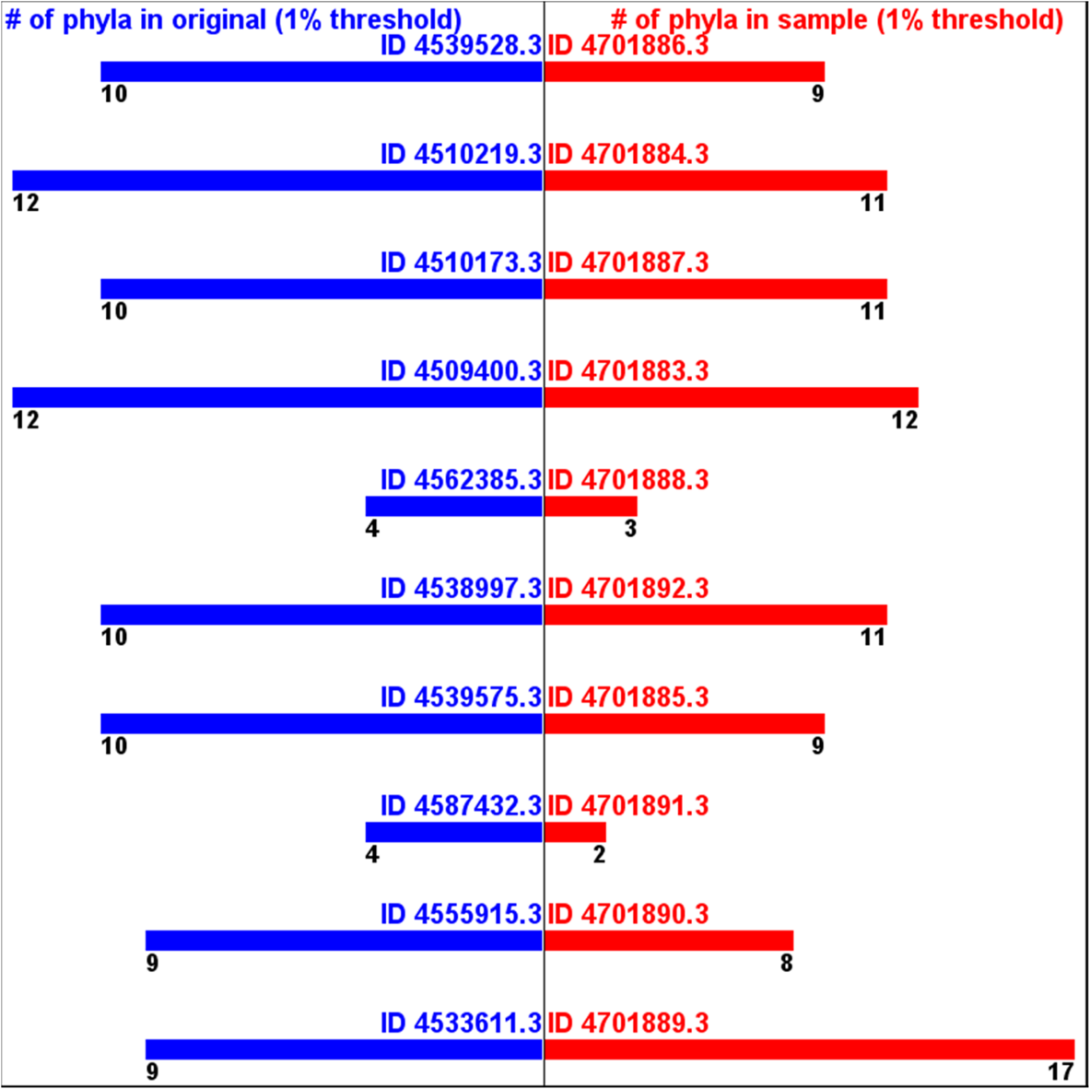
Numbers of annotated phyla (1% threshold) -originals vs. samples

**Fig. 7:**
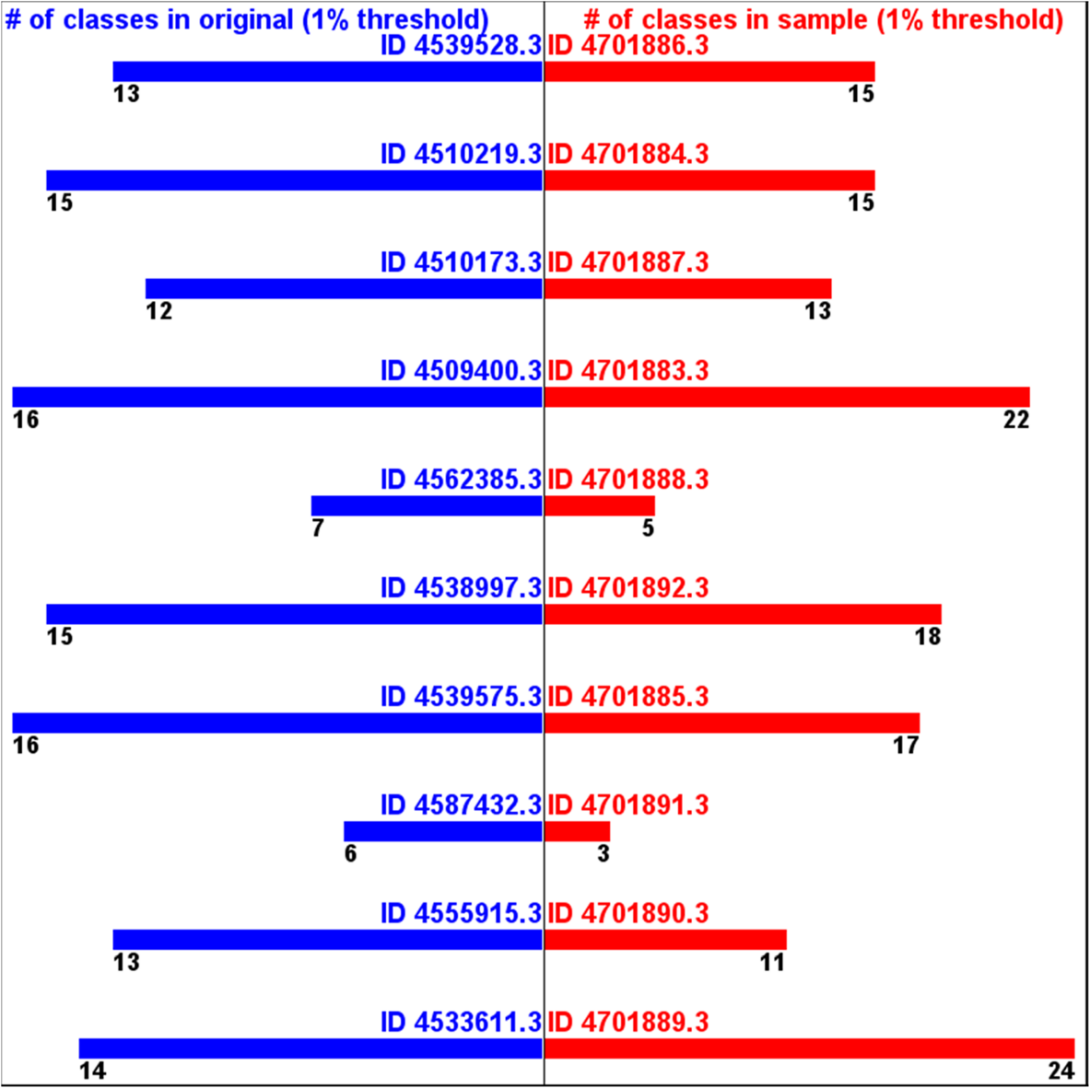
Numbers of annotated classes (1% threshold) -originals vs. samples

However, if we apply 1% threshold to remove over-annotation and/or mis-annotation, the numbers of the annotated phyla from the samples get much closer to the ones from the original data. (Fig. 6) The numbers of the annotated classes from the samples also get closer to the ones from the original data. (Fig. 7) This supports the assumption that this samples still can estimate, at least, part of taxonomic diversity information.

## Discussion

Both the BLAST search tests of the mock community and the results from the publicly downloadable data sets of MG-RAST show that the sampling very small part of sequence data is useful to estimate the brief taxonomic information of the original metagenomic sequences. The sample sequences with their sizes of only 5000 reads, selected from the large sequence data from the existing public cases of MG-RAST, give a useful estimation both to a question of what the most annotated phylum/class is and to a question of how diverse phyla/classes are.

On average, the size of the sample is only 0.002% of the original data, in terms of number of bases. This small size reduces computing time in MG-RAST from several months to a few hours.

It means we can get an estimated result of metagenomic sequence analysis quickly even with less computing resources when we use a small part of genome data. This aligns with the conclusions of shallow sequencing and the results of metagenome skimming to do an efficient analysis with less sequencing.

**Fig. 8:**
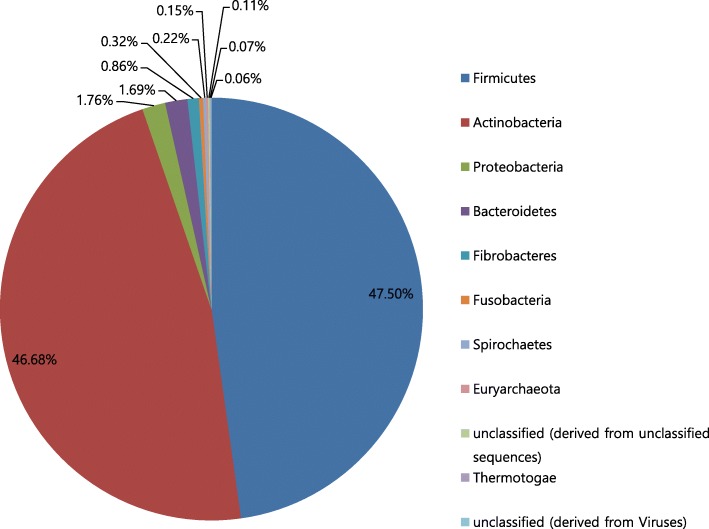
Phyla annotated from original - MG-RAST ID:4587432.3

**Fig. 9:**
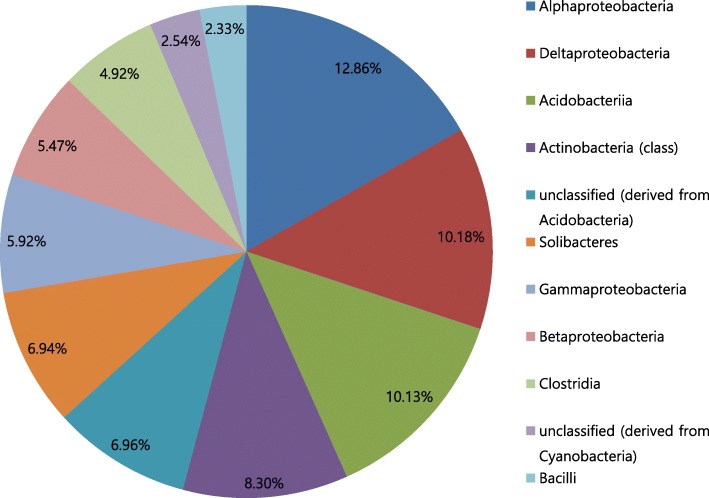
Classes annotated from original MG-RAST ID:4538997.3

On the contrary, In the case where the sample estimates the most annotated phylum incorrectly (MG-RAST ID:4587432.3), the difference between the number of the most annotated phylum (Firmicutes) and the number of the second most annotated phylum (Actinobacteria) in the original is only 0.8% point. (Fig. 8) This small difference is the reason why the estimation from the sample is incorrect. Similarly, in the case where the sample estimates the most annotated class incorrectly (MG-RAST ID:4538997.3), the difference between the number of the most annotated class (Alphaproteobacteria) and the number of the second most annotated class (Deltaproteobacteria) in the original is also as small as 2.2% point. (Fig. 9) These can be regarded as statistical errors. It means an analysis from a sample cannot identify a difference that is smaller than a certain statistical limit.

There is also possibility that the sampling method used here was not the optimal choice. Since our choice of the sampling method was just for minimizing the sampling cost, ignoring quality difference of different sampling methods. If we had tried any pre-checks for different sampling methods, such as comparing GC content values from different sampling methods with GC content of the original data, and tried to find a better sampling method among them, then it could have decreased the error.

On the other hand, the results of the most annotated phyla from MG-RAST tests are Proteobacteria for 8 out of 10 cases and the set of the most annotated classes has only 3 different classes. This is because the metagenomics research data we tested here were chosen only by their original sequence sizes, without any consideration of covering the studies of various phyla and classes. More tests for different metagenomics studies covering more divergent environment might be necessary.

In addition, tests of taxonomic annotation not only for phyla and classes but also for genus and species need to be followed in order to know the applicability of this sampling approach better. Another type of sequence analysis rather than taxonomy annotation needs to be tested, too.

Further quantitative studies to suggest statistical criteria of a sample size, as well as studies of how to apply quality filtering to sample sequences, will also make the approach described here more reliable.

## Conclusions

In spite of this obvious statistical limit, since analysis from a small size sample of metagenomic sequences only takes short time and uses small computing resources, we can still use this approach to develop a standard or a protocol to preview or pre-check metagenomics data, before performing more accurate analysis with original full sequences.

If there is a case where even a brief information of taxonomic distribution is important, we can use estimation by sample to study a biosample much quickly or to study multiple biosamples as many as possible. For example, we can suggest a strategy of metagenomics research, such as analyzing many biosamples quickly or frequently with small samples of sequences, as the first step of screening, and as the second step, analyzing full original sequences of a few biosamples that showed significant characters at the first step.

If we apply this strategy to assessment of soil pollution with bacteria diversity or to assessment of human health with gut microbiota [18], we can screen out unpolluted locations/low risk cases with this quick sample analysis, and can perform more accurate original full sequence analysis only for suspicious locations/cases. We might perform small size sample studies to monitor bacterial diversities of 100 or 1000 spots, covering a whole state or a nation, on a monthly or even on a weekly basis to discover and track environmental change.

Similarly, we can build a strategy to get taxonomic information of bacteria quickly for forensic studies [19, 20] to save time for a criminal investigation. This approach will be also helpful in developing countries where the cost of computing resources is relatively heavy.

## Methods

### Mock community

The mock community with 10 bacteria strains was prepared and their mixed genomes were shotgun sequenced by Hiseq. (Table 4) They are the identical data prepared for a study of Shin S [21].

**Table 4:** Strains of the mock community

- Roseobacter denitrificans OCh114 - Staphylococcus epidermidis ATCC - Polaromonas naphthalenivorans CJ2 - Chromobacterium violaceum ATCC 12472 - Corynebacterium glutamicum ATCC 13032 - Klebsiella pneumoniae KCTC 2242 - Pseudomonas stutzeri ATCC 17588 - Arthrobacter chlorophenolicus A6 - Escherichia coli Strain W - Escherichia coli KCTC 2571	

Then, we selected only small parts from the original full sequences (1,220,000 reads), which were named as “sample sequence set”s or “sample”s. We generated 176 different samples, in total, that are in 11 different sample sizes. For each sample size, we tried 16 different sampling methods. (176 = 11 × 16) The minimum size of the sample was 100 reads (10,100 b) and the maximum size of the sample was 50,000 reads (5,050,000 b).

The sampling methods are categorized as 4 selection types, which are:

1. Selecting the reads from the start, after skipping K number of the reads
2. Selecting the reads from the end, after skipping K number of the reads
3. Selecting the reads from uniformly distributed positions, after skipping K number of the reads
4. Random selection of the reads

We tried 4 different “K number”s for each type. The random selection was tried for 4 different random seeds. Therefore, the 16 different sampling methods applied to each size of the samples.

To review the samples, we calculated GC content, which is one basic way to know the quality of each sample [22].

To get information about taxonomic annotation, we performed a simple BLAST search for the entire sequences of the mock community with respect to the reference genome databases of the strains [23]. BLAST is a widely used software that can search a query sequence out of a reference genome database. Therefore, if there is a given read of metagenome sequences, a researcher can perform a search to know whether it is found as a hit in a reference genome database or not. In this study, BLAST 2.3.0+ was used, with E-value option of 1e-10. The reference genome databases were downloaded from GeneBank [24].

We performed the BLAST search for every single read of the sequences of the mock community with reference genome database for each of all 10 strains. The number of the hits (denoted as *ni*) for genome database of each strain was counted. After all the searches were completed, the sum (denoted as *s*) of all the numbers of the hits counted for all 10 strains was calculated. (*s = Σ ni*) Then, the ratio (*ni*/*s*) of each strain’s hit to the sum was also calculated.

To get the information about the taxonomic annotation from the samples, we, again, performed BLAST search for each sample, in the same way as we did for the original full sequences.

The purpose of this ratio calculation is to do simple comparison between the numbers of the hits from the original full sequences and the numbers of the hits from the samples, not getting the actual information about taxonomic abundance. Therefore, the size difference between reference genomes were not considered.

### MG-RAST

To estimate the brief taxonomic information of the original full sequences, denoted as “original” or “original data” hereafter, in actual cases of bacteria metagenomes, we prepared the samples from the existing research data of metagenomics which were publicly available in MG-RAST [15].

Our purpose was to see how this type of the samples can capture the information of the original data in different real world metagenomics studies. MG-RAST has been a tool allows an external researcher to access the results of existing metagenomics studies already performed before.

We downloaded the sequence files of the public MG-RAST projects with 10 most sequences. (Table 5).

**Table 5:** Public MG-RAST projects with 10 most sequences

Original MG-RAST ID	Project Name	Title of Sequences	Biome	Feature	Material
4,539,528.3	GP corn unassembled	iowa-corn-GAII-round1	terrestrial biome	terrestrial habitat	soil
4,510,219.3	Penang Mangrove Metagenome	BatuMaung_Penang	Mangrove Biome	Wetland	Peat soil
4,510,173.3	BP_Sediments	2011-1933_120131_SN1035_0095_BD04PVACXX_s_4_sequence.fastq	marine benthic biome	ocean	marine sediment
4,509,400.3	Hofmockel Soil Aggregate COB KBASE	PF41-LM-July2012 / H14_ACTTGA_L007	Temperate grasslands	terrestrial habitat	agricultural soil
4,562,385.3	D.I. Tarballs 0610	D.I.Tarball 0610	aquatic biome	mesoscopic physical object	organic material
4,538,997.3	Marcell Experimental Forest carbon cycling	MG-T3F-75cm_pair_retain	Temperate needle-leaf forests or woodlands	forest	soil
4,539,575.3	GED prairie unassembled	1461.5.1405 trimmed	terrestrial biome	terrestrial habitat	soil
4,587,432.3	HMP SRP002423 Bacterial Fungal Taxonomic Analysis	SRS301868_joined	terrestrial biome	human-associated habitat	feces
4,555,915.3	Beijing Hospital Air MetaGenome Part1	ICU-Intensive Care Unit	terrestrial biome	air conditioning unit	air
4,533,611.3		M2_retainMode			

Preparing the selected parts of the originals, in other words, the samples, we selected 5000 reads from the start of each original full sequences. (Table 6) This means we used the sampling method of “selection type 1” and 0 as “K number”.

**Table 6:** Original full sequences and samples from MG-RAST

Original MG-RAST ID	# Of b.p. in Original	# Of Reads in Original	Sample MG-RAST ID	# Of b.p. in Sample	# Of Reads in Sample
4,539,528.3	37,968,936,507	520,346,510	4,701,886.3	306,684	5000
4,510,219.3	56,396,775,865	419,709,973	4,701,884.3	1,011,153	5000
4,510,173.3	37,220,314,566	368,517,966	4,701,887.3	505,000	5000
4,509,400.3	28,875,056,044	285,891,644	4,701,883.3	505,000	5000
4,562,385.3	30,079,534,981	269,568,253	4,701,888.3	749,082	5000
4,538,997.3	36,830,201,101	242,692,675	4,701,892.3	1,179,840	5000
4,539,575.3	19,954,890,565	233,720,367	4,701,885.3	434,006	5000
4,587,432.3	24,845,881,691	220,848,213	4,701,891.3	671,320	5000
4,555,915.3	17,546,603,952	173,728,752	4,701,890.3	505,000	5000
4,533,611.3	17,299,825,549	172,590,841	4,701,889.3	813,458	5000

The only reason why we used this sampling method among other selection types/K numbers was that downloading and handling the entire original sequence data files of these real world studies was too heavy task. If we use selection type 1 and 0 K number, then handling the entire original sequence data files is not necessary. We can just download only the beginning parts of the data files and use them as samples. This reduces sampling cost in terms of data preparation and data handling.

To compare the taxonomic annotation between the originals and the samples, we uploaded the samples to MG-RAST and analyzed them, using MG-RAST, as its available version, that is MG-RAST pipeline 3.6 with its default options. After analysis, MG-RAST gives a list of annotated phyla, classes, and the numbers of how many times they are annotated, as a result.

Since, the original data were from the publicly accessible project, we can also access the analysis results of them, already performed before. Therefore, we can get the lists of annotated phyla, classes and the numbers of how many times they were annotated for the original data, too.

Then, the most annotated phyla from the originals and the most annotated phyla from the samples were compared, as well as the most annotated classes from the originals and the ones from the samples.

To compare taxonomic diversities, the number of the annotated phyla and classes was counted for each of the originals and for each of the samples. Considering over-annotation and/or mis-annotation of MG-RAST, a threshold to ignore phyla and classes with less than 1% hits of the total hits was later applied, in accordance with the study of Peabody et al. [3].

## Additional file

Additional file 1: **Table S1.** The smallest values among the ratios calculated from 16 samples of each sample size (“Ratio from the original” is calculated from the original full sequences, which is same as the value of Table 1). **Table S2.** The largest values among the ratios calculated from 16 samples of each sample size (“Ratio from the original” is calculated from the original full sequences, which is same as the value of Table 1). **Table S3.** Standard deviation from the ratios calculated from 16 samples of each sample size. (DOCX 25 kb)
