# Supplementary material for: What we can see from very small size sample of metagenomic sequences
Source: BMC Bioinformatics. 2018 Nov 3;19:399. doi: 10.1186/s12859-018-2431-8 (PMC6215618; doi:10.1186/s12859-018-2431-8)
Supplement: Supplementary file 1 — Table S1. The smallest values among the ratios calculated from 16 samples of each sample size (“Ratio from the original” is calculated from the original full sequences, which is same as the value of Table 1). Table S2. The largest values among the ratios calculated from 16 samples of each sample size (“Ratio from the original” is calculated from the original full sequences, which is same as the value of Table 1). Table S3. Standard deviation from the ratios calculated from 16 samples of each sample size. (DOCX 25 kb) [file 12859_2018_2431_MOESM1_ESM.docx]

What we can see from very small size sample of metagenomic sequences: Supplement

Table S1 The smallest values among the ratios calculated from 16 samples of each sample size (“Ratio from the original” is calculated from the original full sequences, which is same as the value of Table 1)

| **Size of Sample** | **Escherichia coli KCTC 2571** | **Escherichia coli Strain W** | **Staphylococcus epidermidis ATCC** | **Pseudomonas stutzeri ATCC 17588** | **Klebsiella pneumoniae KCTC 2242** |
| --- | --- | --- | --- | --- | --- |
| **100** | 0.0942 | 0.0942 | 0.0319 | 0.0497 | 0.0484 |
| **200** | 0.1364 | 0.1304 | 0.0743 | 0.0831 | 0.0607 |
| **500** | 0.1476 | 0.1562 | 0.0730 | 0.0888 | 0.0670 |
| **1000** | 0.1630 | 0.1607 | 0.0900 | 0.0956 | 0.0788 |
| **2000** | 0.1631 | 0.1569 | 0.0905 | 0.0996 | 0.0843 |
| **3000** | 0.1664 | 0.1707 | 0.0930 | 0.1014 | 0.0811 |
| **5000** | 0.1684 | 0.1681 | 0.0942 | 0.1025 | 0.0850 |
| **10000** | 0.1776 | 0.1708 | 0.1004 | 0.1044 | 0.0902 |
| **20000** | 0.1785 | 0.1740 | 0.1016 | 0.1054 | 0.0900 |
| **30000** | 0.1807 | 0.1745 | 0.1009 | 0.1073 | 0.0901 |
| **50000** | 0.1805 | 0.1734 | 0.1018 | 0.1071 | 0.0896 |
| **Ratio from the Original** | 0.1862 | 0.1785 | 0.1146 | 0.1127 | 0.0954 |
| **Size of Sample** | **Chromobacterium violaceum ATCC 12472** | **Polaromonas naphthalenivorans CJ2** | **Corynebacterium glutamicum ATCC 13032** | **Roseobacter denitrificans OCh114** | **Arthrobacter chlorophenolicus A6** |
| **100** | 0.0373 | 0.0335 | 0.0205 | 0.0081 | 0.0045 |
| **200** | 0.0564 | 0.0620 | 0.0269 | 0.0439 | 0.0040 |
| **500** | 0.0707 | 0.0547 | 0.0430 | 0.0428 | 0.0061 |
| **1000** | 0.0732 | 0.0714 | 0.0467 | 0.0440 | 0.0098 |
| **2000** | 0.0801 | 0.0763 | 0.0524 | 0.0469 | 0.0071 |
| **3000** | 0.0832 | 0.0756 | 0.0522 | 0.0482 | 0.0085 |
| **5000** | 0.0868 | 0.0765 | 0.0544 | 0.0484 | 0.0107 |
| **10000** | 0.0841 | 0.0783 | 0.0565 | 0.0530 | 0.0095 |
| **20000** | 0.0874 | 0.0812 | 0.0583 | 0.0536 | 0.0118 |
| **30000** | 0.0885 | 0.0818 | 0.0582 | 0.0543 | 0.0122 |
| **50000** | 0.0893 | 0.0822 | 0.0590 | 0.0543 | 0.0001 |
| **Ratio from the Original** | 0.0939 | 0.0855 | 0.0629 | 0.0567 | 0.0137 |
|  |  |  |  |  |  |
|  |  |  |  |  |  |

Table S2 The largest values among the ratios calculated from 16 samples of each sample size (“Ratio from the original” is calculated from the original full sequences, which is same as the value of Table 1)

| **Size of Sample** | **Escherichia coli KCTC 2571** | **Escherichia coli Strain W** | **Staphylococcus epidermidis ATCC** | **Pseudomonas stutzeri ATCC 17588** | **Klebsiella pneumoniae KCTC 2242** |
| --- | --- | --- | --- | --- | --- |
| **100** | 0.2340 | 0.2419 | 0.2971 | 0.1507 | 0.1809 |
| **200** | 0.2045 | 0.2134 | 0.2136 | 0.1614 | 0.1489 |
| **500** | 0.2009 | 0.1994 | 0.1986 | 0.1479 | 0.1306 |
| **1000** | 0.2185 | 0.2050 | 0.1719 | 0.1367 | 0.1148 |
| **2000** | 0.2002 | 0.1976 | 0.1605 | 0.1242 | 0.1125 |
| **3000** | 0.1971 | 0.1916 | 0.1612 | 0.1238 | 0.1056 |
| **5000** | 0.2016 | 0.1877 | 0.1510 | 0.1256 | 0.1109 |
| **10000** | 0.1922 | 0.1862 | 0.1385 | 0.1196 | 0.0993 |
| **20000** | 0.1928 | 0.1855 | 0.1374 | 0.1187 | 0.0976 |
| **30000** | 0.1948 | 0.1836 | 0.1397 | 0.1168 | 0.0997 |
| **50000** | 0.1907 | 0.1826 | 0.1426 | 0.1159 | 0.0992 |
| **Ratio from the Original** | 0.1862 | 0.1785 | 0.1146 | 0.1127 | 0.0954 |
| **Size of Sample** | **Chromobacterium violaceum ATCC 12472** | **Polaromonas naphthalenivorans CJ2** | **Corynebacterium glutamicum ATCC 13032** | **Roseobacter denitrificans OCh114** | **Arthrobacter chlorophenolicus A6** |
| **100** | 0.1840 | 0.1250 | 0.1121 | 0.1346 | 0.0345 |
| **200** | 0.1550 | 0.1157 | 0.0922 | 0.0848 | 0.0409 |
| **500** | 0.1329 | 0.1054 | 0.0820 | 0.1014 | 0.0247 |
| **1000** | 0.1200 | 0.0928 | 0.0724 | 0.0859 | 0.0174 |
| **2000** | 0.1071 | 0.0949 | 0.0846 | 0.0710 | 0.0205 |
| **3000** | 0.1034 | 0.0987 | 0.0792 | 0.0686 | 0.0220 |
| **5000** | 0.1000 | 0.0968 | 0.0716 | 0.0648 | 0.0159 |
| **10000** | 0.0971 | 0.0915 | 0.0694 | 0.0616 | 0.0162 |
| **20000** | 0.0984 | 0.0883 | 0.0692 | 0.0596 | 0.0153 |
| **30000** | 0.0956 | 0.0885 | 0.0675 | 0.0606 | 0.0152 |
| **50000** | 0.0964 | 0.0880 | 0.0665 | 0.0597 | 0.0154 |
| **Ratio from the Original** | 0.0939 | 0.0855 | 0.0629 | 0.0567 | 0.0137 |

Table S3 Standard deviation from the ratios calculated from 16 samples of each sample size

| **Size of Sample** | **Escherichia coli KCTC 2571** | **Escherichia coli Strain W** | **Staphylococcus epidermidis ATCC** | **Pseudomonas stutzeri ATCC 17588** | **Klebsiella pneumoniae KCTC 2242** |
| --- | --- | --- | --- | --- | --- |
| **100** | 0.0389 | 0.0404 | 0.0764 | 0.0313 | 0.0359 |
| **200** | 0.0214 | 0.0240 | 0.0458 | 0.0234 | 0.0259 |
| **500** | 0.0157 | 0.0148 | 0.0318 | 0.0162 | 0.0186 |
| **1000** | 0.0154 | 0.0114 | 0.0238 | 0.0095 | 0.0100 |
| **2000** | 0.0115 | 0.0096 | 0.0183 | 0.0074 | 0.0081 |
| **3000** | 0.0093 | 0.0067 | 0.0158 | 0.0061 | 0.0070 |
| **5000** | 0.0103 | 0.0067 | 0.0159 | 0.0064 | 0.0056 |
| **10000** | 0.0041 | 0.0044 | 0.0128 | 0.0050 | 0.0031 |
| **20000** | 0.0040 | 0.0036 | 0.0118 | 0.0034 | 0.0021 |
| **30000** | 0.0034 | 0.0028 | 0.0127 | 0.0027 | 0.0027 |
| **50000** | 0.0028 | 0.0028 | 0.0127 | 0.0022 | 0.0027 |
| **Size of Sample** | **Chromobacterium violaceum ATCC 12472** | **Polaromonas naphthalenivorans CJ2** | **Corynebacterium glutamicum ATCC 13032** | **Roseobacter denitrificans OCh114** | **Arthrobacter chlorophenolicus A6** |
| **100** | 0.0357 | 0.0202 | 0.0230 | 0.0330 | 0.0087 |
| **200** | 0.0277 | 0.0177 | 0.0175 | 0.0094 | 0.0095 |
| **500** | 0.0169 | 0.0123 | 0.0122 | 0.0144 | 0.0048 |
| **1000** | 0.0129 | 0.0063 | 0.0080 | 0.0098 | 0.0021 |
| **2000** | 0.0076 | 0.0051 | 0.0075 | 0.0068 | 0.0031 |
| **3000** | 0.0058 | 0.0053 | 0.0066 | 0.0063 | 0.0037 |
| **5000** | 0.0041 | 0.0052 | 0.0049 | 0.0037 | 0.0013 |
| **10000** | 0.0036 | 0.0039 | 0.0031 | 0.0023 | 0.0014 |
| **20000** | 0.0028 | 0.0018 | 0.0031 | 0.0017 | 0.0010 |
| **30000** | 0.0021 | 0.0017 | 0.0025 | 0.0015 | 0.0008 |
| **50000** | 0.0019 | 0.0015 | 0.0024 | 0.0016 | 0.0063 |
